# Small-angle neutron scattering applied to low-dose neutron-irradiated Fe–Cr alloys and ferritic martensitic steel Eurofer97^1^

**DOI:** 10.1107/S1600576722004800

**Published:** 2022-06-15

**Authors:** Andreas Ulbricht, André Heinemann, Frank Bergner

**Affiliations:** a Institute of Resource Ecology, Helmholtz-Zentrum Dresden-Rossendorf, Bautzner Landstrasse 400, 01328 Dresden, Germany; b German Engineering Materials Science Centre (GEMS), Heinz Maier-Leibnitz Zentrum (MLZ), Helmholtz-Zentrum Geesthacht GmbH, D-85748 Garching, Germany; University of Luxembourg

**Keywords:** small-angle neutron scattering, Fe–Cr alloys, ferritic martensitic steel, neutron irradiation

## Abstract

Ferritic/martensitic (F/M) Fe–Cr-based steels are candidates for applications in nuclear fission and fusion. Previous experimental results for neutron-irradiated binary Fe–Cr alloys and high-dose neutron-irradiated F/M steels contributed greatly to the understanding of the irradiation behaviour of these groups of materials. However, some details still need to be addressed. Such gaps are related to the effect of secondary alloying and impurity elements, such as Ni and Si, as well as the dose dependence at lower neutron doses [*e.g.* in the range 0.1–1 displacements per atom (dpa)]. This input is essential, for example, for multiscale modelling of irradiation effects or the evaluation of nuclear fission or fusion components at the first stages of operation. Using small-angle neutron scattering, three issues are addressed: (1) the effect of Cr undersaturation (5% Cr) and supersaturation (14% Cr) on the formation of irradiation-induced solute atom clusters/precipitates in low-dose neutron-irradiated Fe–Cr alloys in the presence of intentionally added levels of Ni, Si and P; (2) the effect of irradiation temperature (290°C versus 450°C); and (3) the effect of neutron dose in the range 0.06–0.6 dpa on the irradiation response of the reduced-activation F/M 9%Cr steel Eurofer97. The irradiation-enhanced formation of Cr-rich α′-phase particles was found to be the dominant effect for supersaturated Fe–14Cr–NiSiP at both irradiation temperatures. In contrast, α′ formation is impossible in Fe–5Cr–NiSiP, for which the pronounced irradiation effects observed at 0.1 dpa are mainly attributed to added Ni, Si and P. Finally, Eurofer97 exhibits an exceptionally weak irradiation effect at low neutron doses, the reasons for which are also considered.

## Abstract 2

Characteristics of irradiation-induced nanofeatures derived from magnetic small-angle neutron scattering are reported for low-dose neutron-irradiated Fe–(5–14)Cr–NiSiP model alloys and the reduced-activation ferritic/martensitic 9Cr steel Eurofer97.

## 1. Introduction

Ferritic/martensitic (F/M) Cr steels are candidate materials for fission and fusion applications. Fe–Cr alloys, optionally also containing other elements, serve as model alloys with the aim of deepening the understanding of neutron irradiation effects. Irradiation-induced nanofeatures in neutron-irradiated Fe–Cr alloys of different Cr contents have been systematically studied using several experimental techniques. These include transmission electron microscopy (TEM) (Matijasevic & Almazouzi, 2008 ▸; Hernández-Mayoral *et al.*, 2016 ▸), atom probe tomography (APT) (Kuksenko *et al.*, 2011 ▸, 2013*a* ▸,*b* ▸), magnetic (Heintze *et al.*, 2011 ▸) and nuclear (Heintze *et al.*, 2010 ▸) small-angle neutron scattering (SANS), and positron annihilation spectroscopy (Lambrecht & Malerba, 2011 ▸). New insight was gained from the reported results, in brief: (1) on the formation of dislocation loops as a function of Cr and the absence or presence of nanovoids under the applied irradiation conditions (Matijasevic & Almazouzi, 2008 ▸; Hernández-Mayoral *et al.*, 2016 ▸); (2) on the irradiation-enhanced formation of Cr-rich α′-phase particles, which also gave fresh impetus to the revision of details of the Fe–Cr binary phase diagram (Bonny *et al.*, 2008 ▸; Bergner *et al.*, 2009 ▸; Xiong *et al.*, 2010 ▸); and (3) on the role of impurity elements such as Ni, Si and P in the formation of solute-rich clusters (SRCs) (Kuksenko *et al.*, 2011 ▸, 2013*a*, ▸ *b* ▸). It was also found that (4) the consideration of dislocation loops, α′-phase particles and SRCs was necessary and sufficient to explain the measured irradiation-induced hardness increase (Bergner *et al.*, 2014 ▸). The observation of Cr–Ni–Si–P-enriched SRCs motivated the fabrication and irradiation of a set of alloys (Konstantinović & Malerba, 2020 ▸) containing 5, 9 and 14% Cr as well as balanced amounts of Ni, Si and P, both separately and in combination. A systematic characterization of the irradiated alloys using the methods mentioned is not yet complete. The first results indicate the formation of dislocation loops and the absence of TEM-visible nanovoids in Fe–9Cr–NiSiP (Dubinko *et al.*, 2020 ▸). The present study is focused on the alloys containing Ni, Si and P in combination and the application of (mainly magnetic) SANS. The irradiation conditions cover a dose of approximately 0.1 displacements per atom (dpa) and irradiation temperatures of 290 and 450°C. Note that 5% Cr and 14% Cr correspond to Cr undersaturation and supersaturation, respectively, in the binary Fe–Cr system for both temperatures. Application of SANS to this set of ferromagnetic alloys with the samples exposed to a saturation magnetic field can separate magnetic and nuclear scattering contributions, allowing derivation of size distributions of irradiation-induced nanofeatures by subtracting the unirradiated reference, and revealing an indicator (called the *A* ratio) of the type of nanofeature.

As mentioned previously, Fe–Cr(–*X*)-based model alloys serve as model systems for F/M steels. A special reduced-activation variant, Eurofer97, is a candidate material for nuclear fusion applications. Early SANS investigations of neutron-irradiated F/M steels reported by Mathon *et al.* (2003 ▸) revealed the phenomenon of irradiation-enhanced α′-phase formation. Neutron-irradiated Eurofer97 has been studied using several methods including TEM (*e.g.* Matijasevic *et al.*, 2008 ▸; Klimenkov *et al.*, 2011 ▸; Dethloff *et al.*, 2016 ▸), APT (Rogozhkin *et al.*, 2013 ▸; Gómez-Ferrer *et al.*, 2020 ▸) and SANS (Coppola *et al.*, 2009 ▸, 2018 ▸; Coppola & Klimenkov, 2019 ▸). The most prominent TEM-visible irradiation-induced defects at low doses (about 1 dpa or less) and low irradiation temperatures (about 300°C or less) are dislocation loops (Matijasevic *et al.*, 2008 ▸). Nanovoids were not found by TEM under these conditions (Matijasevic *et al.*, 2008 ▸). Higher neutron doses, higher irradiation temperatures (Klimenkov *et al.*, 2020 ▸) and transmutational helium (Dethloff *et al.*, 2016 ▸) promote the formation of TEM-visible nanovoids or helium bubbles. APT studies of neutron-irradiated (15 and 32 dpa, 330°C) Eurofer97 revealed the formation of Cr–Mn(–Si)-enriched SRCs (Rogozhkin *et al.*, 2013 ▸; Gómez-Ferrer *et al.*, 2020 ▸) and Si–P–Ni–Mn–Cr-rich ‘regions’ (Gómez-Ferrer *et al.*, 2020 ▸). In the present SANS study, we extend the database to cover lower-dose irradiations at 290°C including irradiations up to 0.06, 0.1 and 0.6 dpa. For assessment and interpretation of the results, we benefit from the insight gained for the Fe–*x*Cr(–NiSiP) model alloys.

## 2. Experiments

### 2.1. Materials and irradiation conditions

The first part of this study is dedicated to Fe–Cr-based model alloys nominally containing 5, 9 and 14 mass% Cr as well as defined levels of Ni, Si and P. The alloy compositions are specified in Table 1 ▸. Details related to fabrication and processing are reported by Konstantinović & Malerba (2020 ▸). The 5Cr and 9Cr alloys exhibit ferritic microstructures (ferrite grain sizes approximately 30 µm) and contain islands of bainite of negligible volume fraction. For the dislocation density of Fe–9Cr–NiSiP, a value of 1.4 × 10^13^ m^−2^ was reported (Dubinko *et al.*, 2020 ▸). The 14Cr alloy is fully ferritic with a grain size of 240 µm.

**Figure 1:**
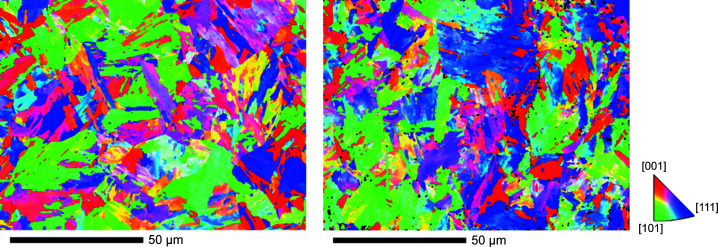
EBSD IPF-X maps of Eurofer97 heats A (left) and B (right) with the colour code indicated.

As a second part, two heats of the 9Cr reduced-activation ferritic/martensitic steel Eurofer97, also referred to as 9Cr1WVTi, are studied. Heat E83699 (8.99 mass% Cr), referred to below as A, was produced as a forged bar of 100 mm diameter, austenitized at 980°C in air and tempered at 740°C in air. Heat E83698 (8.82 mass% Cr), referred to below as B, was produced as a rolled plate of 14 mm thickness, austenitized at 980°C in air and tempered at 760°C in air. The compositions of heats A and B are specified in Table 2 ▸. For more details on fabrication and processing, see the work by Rieth *et al.* (2003 ▸). Both steels are martensitic. Inverse pole figure (IPF-X) maps obtained by electron backscatter diffraction (EBSD) are shown in Fig. 1 ▸ to illustrate the finely subdivided martensitic microstructure for Eurofer97. The average EBSD grain size was measured to be 2.2 and 2.1 µm for heats A and B, respectively. TEM micrographs also revealing evidence on the types of carbides were shown by Fernández *et al.* (2001 ▸, 2002 ▸) and Matijasevic *et al.* (2008 ▸). A dislocation density of 8 × 10^13^ m^−2^ was reported for heat A (Matijasevic *et al.*, 2008 ▸).

The samples of 10 mm diameter and 1 mm thickness were exposed to neutron irradiation at temperatures of 290 and 450°C in the BR2 materials testing reactor of SCK·CEN. A detailed description of the irradiation experiment is provided by Konstantinović & Malerba (2020 ▸). The neutron flux was approximately 1.5 × 10^13^ cm^−2^ s^−1^ (*E* > 1 MeV), and the resulting displacement damage was 0.11 dpa. Additional samples of Eurofer97 (A) (size 7 × 7 × 1 mm) irradiated earlier at 290°C up to 0.06 and 0.6 dpa (Matijasevic *et al.*, 2008 ▸) were also studied. Eurofer97 (B) was only investigated under the unirradiated conditions using a sample size of 10 × 10 × 1 mm.

### 2.2. SANS experiments and Vickers hardness testing

The SANS experiments reported in this study were conducted at the beamline V4 of the Helmholtz-Zentrum Berlin (HZB) and at the instrument SANS-1 of the Heinz Maier-Leibnitz Zentrum Garching (MLZ). In the former experiment, the neutron wavelength λ was 0.6 nm and the sample–detector distances were 1.7 and 8 m with corresponding collimation lengths. The latter experiment was carried out using a neutron wavelength of 0.45 nm, sample–detector distances of 1.6 and 8 m, and collimation lengths of 4 and 8 m. Saturation magnetic fields of approximately 1.5 T oriented perpendicular to the neutron beam were applied to the samples in both experiments. For absolute calibration, a poly(methyl methacrylate) sample (detector efficiency) and a reference sample with well known differential magnetic and nuclear scattering cross sections were measured. Data reduction including separation of magnetic and nuclear contributions was carried out using the *BerSANS* software package (Keiderling, 2002 ▸).

For the basics of SANS, the analysis of SANS data and typical applications in materials science, we refer the reader to the literature (Kostorz, 1979 ▸; Fratzl, 2003 ▸; Mühlbauer *et al.*, 2019 ▸). In order to calculate the size distribution of scatterers, a dilute two-phase matrix-inclusion microstructure composed of homogeneous spherical scatterers (with a sharp interface) randomly dispersed in an otherwise homogeneous matrix was assumed. For a distribution of spherical scatterers of radius *R* in the probed volume, the coherent magnetic scattering cross section dΣ_mag_/dΩ can be expressed as

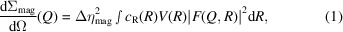

with the scattering vector magnitude *Q* = (4π/λ)sin(θ/2) (θ is the scattering angle); the form factor

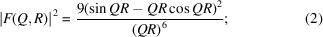

the volume of the sphere *V*(*R*) = 4π*R* ^3^/3; and the sought volume fraction per size increment *c*_R_. The magnetic contrast 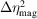 is the squared difference between the magnetic scattering length densities of the scatterer and the matrix (Mühlbauer *et al.*, 2019 ▸). Equation (1) is also applicable to the nuclear scattering cross section (replace subscript ‘mag’ with subscript ‘nuc’). In order to calculate the nuclear contrast 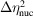 , the structure and composition of the scatterers and the matrix have to be known. This option was not used in the present study focused on magnetic SANS. Instead, the volume fractions and size distributions of scatterers were derived from the magnetic scattering cross sections on the assumption of non-magnetic scatterers in the respective ferromagnetic Fe–Cr matrix. This yields a contrast 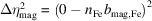 , with the atomic density of the Fe atoms in the body-centered cubic (b.c.c.) Fe–Cr matrix *n*_Fe_ = 84.6 nm^−3^, and the magnetic scattering length of Fe *b*_mag, Fe_ = 6 fm. For Fe containing 0, 5, 9 and 14% Cr, the resulting values of the magnetic contrast are 2.57, 2.32, 2.13 and 1.90 [in units of cm^−1^ nm^−3^ for appropriate use in equation (1)], respectively. Note that we do not know the real magnetic scattering length density of the scatterers in advance. In the case of a nonzero value, our assumption will give rise to a systematic underestimation of the real volume fraction of scatterers (Mühlbauer *et al.*, 2019 ▸). We will revisit this issue in the *Discussion* [Sec sec4]. We are only interested in the family of irradiation-induced scatterers. To separate this family from pre-existing scatterers, the scattering curves of the respective unirradiated references were subtracted from the scattering curves of the irradiated samples on the left-hand side of equation (1). The size distribution of irradiation-induced scatterers was then estimated by solving the inverse problem using the indirect Fourier transform method introduced by Glatter (1980 ▸).

To calculate the *A* ratio [first introduced by Frisius & Buenemann (1979 ▸)] of the irradiation-induced scatterers, we used the following identities:

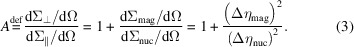

Subscripts 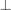 and 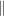 indicate scattering perpendicular and parallel to the magnetic field direction, respectively. Again, the unirradiated reference has to be subtracted beforehand. The calculation of the theoretical *A* ratio as a signature of the type of scatterers is based on the assumption that the magnetic and nuclear scatterers are the same objects.

The experiments covered the characterization of irradiation-induced nanofeatures for the 290°C irradiations (all Fe–Cr alloys and heat A of Eurofer97) and the 450°C irradiations (Fe–5Cr–NiSiP and Fe–14Cr–NiSiP only) along with the unirradiated reference samples. Results obtained for the Fe–9Cr–NiSiP alloy irradiated at 290°C were already reported before in another context (Konstantinović *et al.*, 2020 ▸). The Vickers hardness HV10 (load 98.1 N) was measured after completion of the SANS experiments by averaging over ten individual hardness tests for each sample.

## 3. Results

### 3.1. Fe–Cr–NiSiP

**Figure 2:**
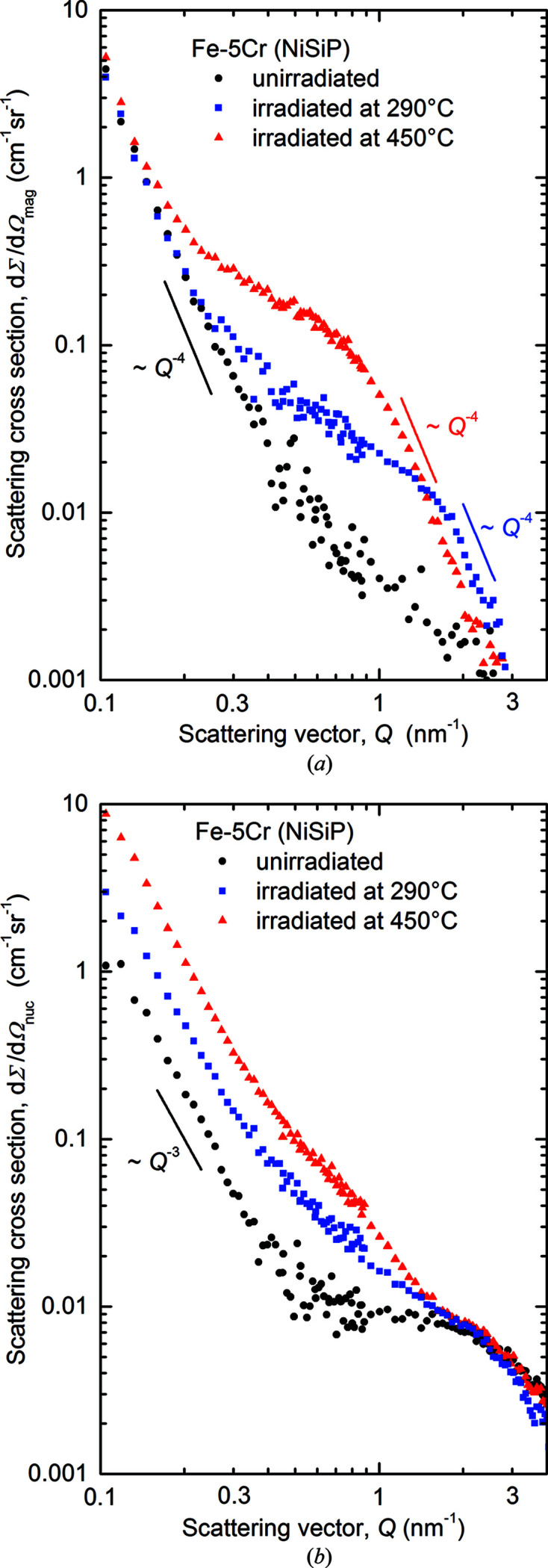
Separated magnetic (*a*) and nuclear (*b*) scattering cross sections for Fe–5Cr–NiSiP.

The separated magnetic and nuclear scattering cross sections as functions of the scattering vector magnitude *Q* are plotted in Figs. 2 ▸ and 3 ▸ for Fe–5Cr–NiSiP and Fe–14Cr–NiSiP, respectively. Scattering curves for the neutron-irradiated (290 and 450°C) samples and the unirradiated references are shown. Asymptotic power-law exponents are indicated. For the results for Fe–9Cr–NiSiP (solely 290°C), we refer to Konstantinović *et al.* (2020 ▸).

The magnetic scattering curves obtained for Fe–5Cr–NiSiP [Fig. 2(*a* ▸)] represent the expected asymptotic behaviour as frequently observed in previous work (*e.g.* Heintze *et al.*, 2011 ▸) for Fe–Cr alloys. For the unirradiated condition, the curve follows the *Q* ^−4^ dependence known as the Porod law (Kostorz, 1979 ▸) at least down to *Q*_min_ = 0.1 nm^−1^. This is attributed to the presence of scatterers much larger than 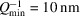 . Both irradiated conditions cover the transition from the Guinier behaviour (where the curves come close to the unirradiated reference) to the Porod (*Q* ^−4^) behaviour, indicating the presence of irradiation-induced scatterers smaller than 10 nm (see below).

The nuclear scattering curves for Fe–5Cr–NiSiP exhibit deviations from the ‘ideal’ behaviour outlined above. At lower *Q* values (0.1–0.5 nm^−1^), the nuclear scattering for the irradiated samples and the unirradiated reference follows a *Q* ^−3^ dependence. The irradiations give rise to shifts towards larger scattering cross sections. This behaviour does not find its equivalent in magnetic scattering and vice versa, indicating that the dominant magnetic and nuclear scatterers are different objects. Hence, the *A* ratio as an indicator of the type of scatterers is meaningless in this case. In the present study, we are mainly focused on magnetic scattering.

For Fe–14Cr–NiSiP, the magnetic and nuclear scattering curves are sufficiently close to the ‘ideal’ behaviour for the standard analysis to be applied. The intermediate increase of the scattering cross sections of the irradiated samples as a function of *Q* indicates interparticle interference effects typical of concentrated systems. This requires special attention in the analysis below.

In Figs. 4 ▸ and 5 ▸, the magnetic difference scattering curves with the unirradiated samples taken as reference (*a*) and the reconstructed size distributions (*b*) are displayed, again for Fe–5Cr–NiSiP and Fe–14Cr–NiSiP, respectively. Non-magnetic scatterers in the ferromagnetic matrix were assumed for scaling of the size distributions and volume fractions. It is important to reiterate that this assumption may result in an underestimation of the volume fraction if the scatterers bear a nonzero magnetic scattering length density. The fit curves shown are the Fourier counterparts of the reconstructed size distributions of irradiation-induced scatterers plotted in Figs. 4(*b*) ▸ and 5(*b*) ▸. The quality of the fits underpins the validity of the size distributions. For the 14Cr alloy, only the *Q* ranges beyond the positions of the maxima were fitted. As a consequence, the reconstructed size distributions are less accurate compared with cases with availability of the entire *Q* range. However, the concept of the Porod invariant (Porod, 1982 ▸) remains valid for concentrated systems; it was applied to obtain improved estimates of the total volume fraction (Bergner *et al.*, 2009 ▸).

The measured *A* ratios of irradiation-induced scatterers in Fe–14Cr–NiSiP are plotted in Fig. 6 ▸ as functions of *Q*. The *A* ratio is not shown for Fe–5Cr–NiSiP, because the magnetic and nuclear scatterers appear to be different objects, which does not allow appropriate interpretation within our framework.

The estimated total volume fractions of irradiation-induced scatterers (mainly SRCs or α′-phase particles, see *Discussion* [Sec sec4]), mean radii and average *A* values are summarized in Table 3 ▸ along with the measured values of the Vickers hardness increase. Results for Fe–9Cr–NiSiP reported by Konstantinović *et al.* (2020 ▸) are included in order to additionally underpin the discussion of the Cr effect.

### 3.2. Eurofer97

The magnetic and nuclear scattering cross sections derived for the unirradiated conditions of the two heats of Eurofer97 are plotted in Fig. 7 ▸. In the lower *Q* range (0.1–0.5 nm^−1^), the scattering curves are in agreement for heats A and B and follow the Porod law. At higher *Q*, the cross sections derived for heat B are larger for both the magnetic and the nuclear case.

The magnetic and nuclear scattering cross sections derived for the 0.6 dpa irradiation of heat A are compared in Fig. 8 ▸ with the unirradiated reference. The results indicate a small irradiation-induced increase in the range *Q* > 0.5 nm^−1^.

The format of the scattering plots shown in Fig. 8 ▸ is not well suited to visualizing the smallest irradiation-induced changes. Such changes are better highlighted in terms of difference scattering curves. Fig. 9 ▸(*a*) summarizes the magnetic difference scattering curves and fits derived for the three irradiation conditions of Eurofer97 (heat A). In order to compare the irradiation effect with a possible effect of the fabrication process, the measured difference between the scattering cross sections obtained for heats A and B (compare Fig. 7 ▸) is included. Moreover, we also provide the magnetic difference scattering curve for a binary Fe–9Cr alloy irradiated in the same irradiation experiment as the 0.11 dpa irradiation of Eurofer97 (Konstantinović *et al.*, 2020 ▸). Minor deviations between the fit and data points at low *Q* values are due to coincidental sample-to-sample differences instead of irradiation-induced objects. The size distributions of irradiation-induced scatterers (*i.e.* the inverse Fourier transforms of the difference scattering curve fits) obtained for Eurofer97 (heat A) and binary Fe–9Cr are summarized in Fig. 9 ▸(*b*).

The *Q* dependence of the *A* ratio obtained from the magnetic and nuclear irradiation-induced difference scattering curves is plotted in Fig. 10 ▸ for 0.6 dpa irradiation. The *A* ratio is approximately 2 for the lower *Q* values and increases to approximately 4 for the largest *Q* values. For the 0.06 and 0.11 dpa irradiations, the measured difference cross sections are so small that the errors of the *A* ratio exceed the value of *A*. The average characteristics of the irradiation-induced scatterers in Eurofer97 are collected in Table 4 ▸, underpinned with the measured irradiation-induced increase of Vickers hardness.

## 4. Discussion

### 4.1. Fe–Cr–NiSiP

The first observation to be discussed is the formation of irradiation-induced nanofeatures in the 290 and 450°C irradiations of Fe–5Cr–NiSiP (Figs. 2 ▸ and 4 ▸). Expected types of nanofeatures in this system are SRCs (Kuksenko *et al.*, 2013 ▸ *b*) and dislocation loops (Hernández-Mayoral *et al.*, 2016 ▸; Dubinko *et al.*, 2020 ▸). TEM-visible nanovoids can be tentatively excluded by analogy with Fe–9Cr–NiSiP irradiated under the same conditions (Dubinko *et al.*, 2020 ▸). Cr-rich α′-phase particles can also be excluded, because the Cr content is well below the solubility limit (Xiong *et al.*, 2010 ▸; Bonny *et al.*, 2010 ▸). (Undecorated) dislocation loops (*i.e.* planar interstitial atom clusters) are certainly present but do not contribute to magnetic scattering due to the low scattering contrast (Seeger & Rühle, 1963 ▸; Bergner *et al.*, 2008 ▸), at least as long as scatterers of higher contrast are available. The only remaining type of scatterer is the SRCs. For an irradiation temperature of 450°C, the volume fraction and mean radius of SRCs are slightly larger than for 290°C, consistent with a larger mean diffusion distance of the cluster-forming elements at the higher temperature. The resulting increases of Vickers hardness are in agreement within the error.

An interesting detail of nuclear scattering in Fe–5Cr–NiSiP [Fig. 2 ▸(*b*)] is the *Q* ^−3^ dependence in the *Q* range 0.1–0.5 nm^−1^ with an irradiation-induced shift towards higher scattering cross sections. A *Q* ^−3^ dependence was predicted for nuclear scattering by dislocations (Atkinson & Hirsch, 1958 ▸). We assume, as a working hypothesis, that this behaviour might be due to irradiation-induced segregations of Si and P to extended defects such as line dislocations and grain boundaries. Such segregations have been reported for similar neutron-irradiated Fe–Cr alloys (Kuksenko *et al.*, 2013*a* ▸). The atomistically small dimensions of the segregation fields in one (grain boundary) or two directions (dislocation) may be responsible for the absence of the equivalent of nuclear scattering in magnetic scattering. We must leave this issue for a dedicated future investigation. Conversely, the absence of the equivalent of magnetic scattering in nuclear scattering may be due to a vanishingly small nuclear contrast of the magnetic scatterers (Ni–Si–P-enriched SRCs) with respect to the Fe–5Cr matrix. This is possible even for solute atoms occupying solely b.c.c. Fe lattice positions, because the value of the nuclear scattering length for Ni (*b*_nuc_ = 10.3 fm) is higher than the value for Fe (*b*_nuc_ = 9.45 fm), whereas the values for Si (*b*_nuc_ = 4.15 fm) and P (*b*_nuc_ = 5.13 fm) are smaller (Koester *et al.*, 1991 ▸). Hence, the scattering length densities of scatterer and matrix may coincidentally agree for certain SRC compositions. In any case, if nanovoids were responsible for the observed magnetic scattering, the nuclear equivalent would have to be there, which is not the case. This is another strong confirmation of the absence or very minor contribution of nanovoids in 0.11 dpa neutron-irradiated Fe–5Cr–NiSiP.

**Figure 3:**
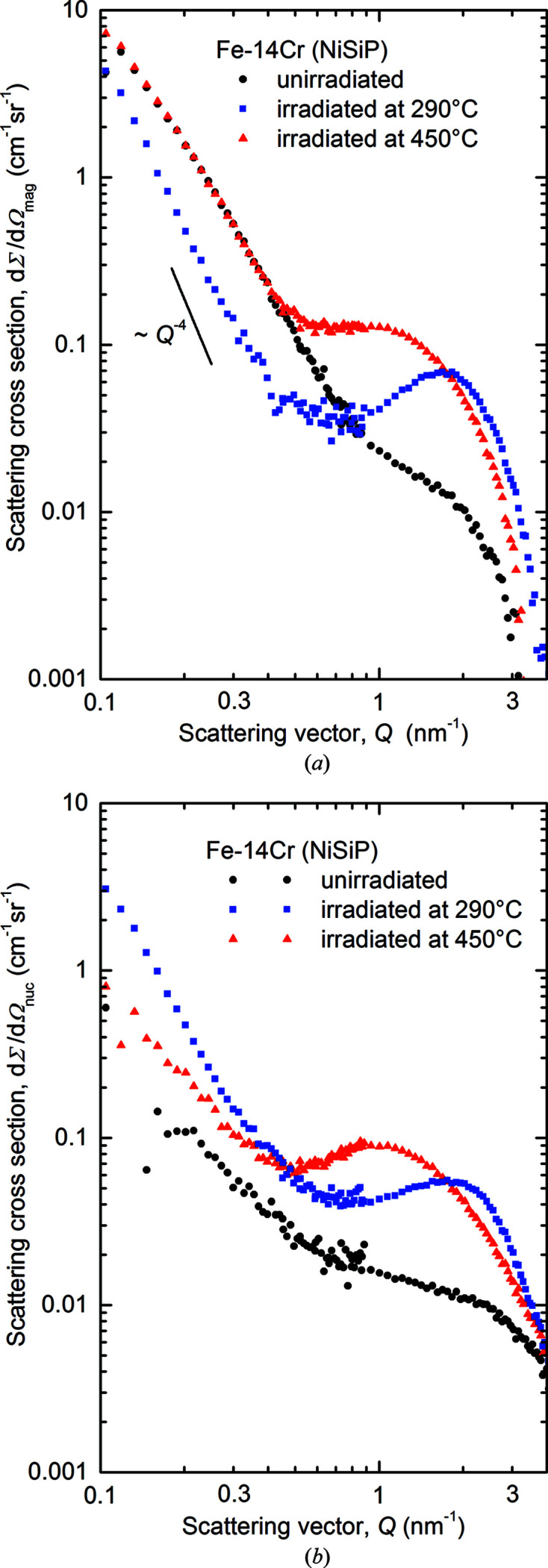
Separated magnetic (*a*) and nuclear (*b*) scattering cross sections for Fe–14Cr–NiSiP.

**Figure 4:**
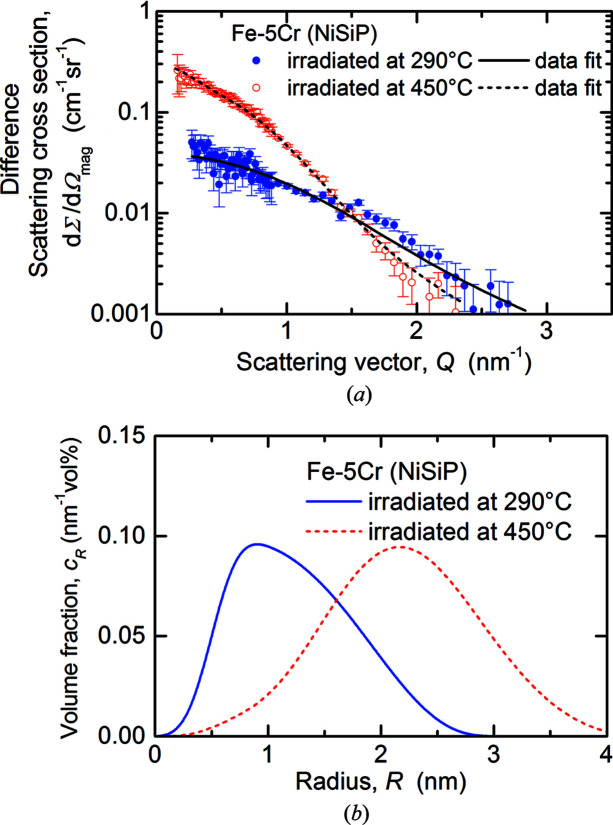
Magnetic difference scattering cross sections with fits (*a*) and size distributions (*b*) for Fe–5Cr–NiSiP.

**Figure 6:**
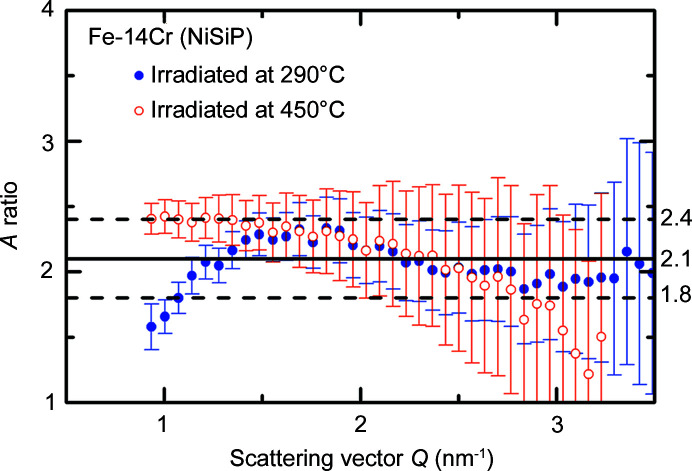
Measured *A* ratio for the difference scattering curves of Fe–14Cr–NiSiP. The line and band indicate the average value and error range of *A*, respectively.

For Fe–14Cr–NiSiP, the most prominent observation is the appearance of minima in the scattering curves for the irradiated conditions in both magnetic and nuclear scattering (Fig. 3 ▸). This is indicative of interference effects occurring in concentrated systems. The Porod invariant, which is equally applicable to dilute and concentrated systems, was utilized to calculate the volume fractions of scatterers. The estimates of the *A* ratio of irradiation-induced scatterers (Table 3 ▸) confirm the dominance of nanometric α′-phase particles. Theoretical *A* ratios of α′ between 1.8 and 2.2 were reported in the literature depending on the assumptions about the atom fractions and magnetic moments of Fe and Cr (Mathon *et al.*, 2003 ▸; Ulbricht *et al.*, 2010 ▸; Tissot *et al.*, 2019 ▸). This agrees reasonably well with the measurements (Table 3 ▸). There is no reason why SRCs reported in the literature (Kuksenko *et al.*, 2013 ▸ *b*) and observed for the irradiated 5Cr alloys (volume fractions of 0.13 and 0.16%) should be absent in the irradiated 14Cr alloys. In any case, the volume fraction of those SRCs should be much less than the total volume fraction (1.75 and 1.1% for 290 and 450°C, respectively).

If we assume the scatterers in the 14Cr alloys to be exclusively α′ as a simplifying approximation, we can compare the measured volume fractions with estimates based on the Fe–Cr equilibrium phase diagram (Bonny *et al.*, 2010 ▸). Application of the lever rule and conversions between atom%, mass% and volume fraction yield estimates of the expected α′ volume fraction of 9.1 and 3.2% at equilibrium temperatures of 290 and 450°C, respectively. The experimental values 1.75 and 1.1% indicate that the process of irradiation-enhanced α′ formation is not yet finished at a neutron dose of 0.11 dpa. In contrast, equilibrium was reportedly reached in an Fe–12Cr alloy at a neutron dose of 0.6 dpa (Bergner *et al.*, 2009 ▸). The higher percentage of the measured fraction of α′ formed owing to the 450°C/0.11 dpa irradiation with respect to the calculated equilibrium fraction (34% compared with 19% for 290°C) again is consistent with the higher Cr diffusivity at 450°C. The same reasoning is also applicable to the significantly larger mean size of scatterers for 450°C (see Table 3 ▸).

The irradiation-induced increase of the Vickers hardness is larger for the 290°C irradiation of the 14Cr alloy, meaning it follows the ranking of the volume fractions of scatterers in the 14Cr samples. Comparing 5Cr and 14Cr alloys, the moderately higher hardness increase for 14Cr, at much higher volume fraction, is a clear consequence of the smaller dimensionless obstacle strength of α′-phase particles for dislocation motion compared with SRCs, namely 0.015 versus 0.1 according to Bergner *et al.* (2014 ▸). The results reported for Fe–9Cr–NiSiP irradiated at 290°C are broadly consistent with the results and interpretation for the 5Cr and 14Cr alloys with the additional comment that the solubility limit of Cr in α is close to 9% Cr (Bonny *et al.*, 2010 ▸).

### 4.2. Eurofer97

A tentative interpretation of the scatterers detected in 0.6 dpa neutron-irradiated Eurofer97 is based on the measured *A* ratio and relevant reported results. The link with much higher neutron doses, for which more reported evidence is available, will be addressed later below. For Eurofer97, the estimated *A* ratio, *A* = 2.2 ± 0.5 for 0.6 dpa, is compatible with the expectation for α′-phase particles (*A* in the range 1.8–2.2, see above). This is broadly consistent with reported results on other neutron-irradiated F/M 9Cr steels (Mathon *et al.*, 2003 ▸) and Fe–9Cr model alloys (Ulbricht *et al.*, 2010 ▸; Kuksenko *et al.*, 2013 ▸ *b*) irradiated under similar conditions. As the solubility limit of Cr in α at 290°C is close to 9% (Bonny *et al.*, 2010 ▸), final confirmation of the formation and amount of α′ in Eurofer97 based on the phase diagram is not practicable. Given the relatively wide error range of the measured *A* ratio, which is a consequence of the weak irradiation effect, SRCs of unknown composition cannot be excluded. Indeed, the Cr–Mn(–Si)-enriched clusters reported for higher doses are candidates (Rogozhkin *et al.*, 2013 ▸; Gómez-Ferrer *et al.*, 2020 ▸). In contrast, the theoretical *A* ratio of nanovoids in Fe–9Cr, *A* = 1.37 according to Ulbricht *et al.* (2010 ▸), is significantly smaller than the measured range of *A*, confirming either the absence or a very minor contribution of nanovoids in 0.6 dpa Eurofer97. This is consistent with the absence of TEM-visible nanovoids in the same heat of Eurofer97 irradiated in the same experiment (Matijasevic *et al.*, 2008 ▸). In the latter study, irradiation-induced dislocation loops were detected, but undecorated loops represent negligibly weak scatterers as mentioned previously for the model alloys. For the 0.11 and 0.06 dpa irradiations, the difference scattering cross sections are too small for a meaningful *A* ratio to be calculated. However, it is reasonable to assume that the conclusions drawn above for 0.6 dpa are also applicable to the scatterers observed for the lower doses.

The volume fraction of scatterers deduced from the magnetic scattering cross sections on the assumption of non-magnetic scatterers in neutron-irradiated Eurofer97 (heat A) is plotted in Fig. 11 ▸ as a function of dose. A power-law dependence often used in the field of neutron embrittlement of reactor pressure vessel steels (Soneda, 2015 ▸; Castin *et al.*, 2020 ▸) was fitted to the SANS data. The resulting curve is shown along with the best-fit equation. The estimated power-law exponent in the dose range 0.06–0.6 dpa is 0.79.

It would be interesting to extend the dose range covered in Fig. 11 ▸ towards higher doses. SANS results for 2.7 (300°C), 8.4 (300°C) and 16.3 dpa (250°C) have been reported in the literature (Coppola *et al.*, 2009 ▸; Coppola & Klimenkov, 2019 ▸). The reported volume fractions were calculated from nuclear scattering cross sections on the assumption that the scatterers were nanovoids. However, the assumption of nanovoids is incompatible with the *A* ratio of the irradiation-induced scatterers reported by the same authors to be approximately 2. This is significantly higher than the theoretical value of 1.37 expected for nanovoids in Fe–9Cr (Ulbricht *et al.*, 2010 ▸). Note, the reported *A* ratio is close to the *A* ratio observed for 0.6 dpa neutron-irradiated Eurofer97 in the present study. In any case, a reconsideration of the reported results in terms of magnetic SANS with the assumption of non-magnetic scatterers in a ferromagnetic matrix would facilitate comparability with the present observations.

Much smaller volume fractions of irradiation-induced scatterers in neutron-irradiated Eurofer97 were found compared with the model alloys. For the same irradiation conditions (290°C, 0.11 dpa), the volume fraction is approximately 15 times smaller than for Fe–9Cr–NiSiP. There are two different reasons contributing to this high irradiation resistance: first, the impurity levels of Ni (<0.007 mass%), Si (<0.07 mass%) and P (0.004 mass%) in Eurofer97 (heat A) (Table 2 ▸) are much smaller compared with the respective contents in the Fe–Cr–NiSiP model alloys (Table 1 ▸). Therefore, SRCs cannot form at comparable amounts. Second, the specific grain boundary area (grain size 2.2 µm versus 30 µm), the dislocation density (8 × 10^13^ m^−2^ versus 1.4 × 10^13^ m^−2^) and the specific carbide interface area (high density of carbides versus no carbides) of the unirradiated martensitic steel are much larger than for the ferritic Fe–9Cr model alloy. This results in a higher sink strength for irradiation-induced point defects in Eurofer97 (Was, 2007 ▸; Duan *et al.*, 2017 ▸); more point defects are lost at sinks under steady-state irradiation conditions. Therefore, fewer point defects are available for the irradiation-enhanced diffusion of alloying/impurity elements needed to form irradiation-induced solute clusters that harden and embrittle the steel (Castin *et al.*, 2020 ▸). The decisive role of the sink strength is confirmed by comparing the results obtained for 0.11 dpa Eurofer97 with results reported for pure Fe–9Cr (same dose) (Konstantinović *et al.*, 2020 ▸). The volume fraction estimated for Eurofer97 is still 8 times smaller than the volume fraction estimated for pure Fe–9Cr (0.15%). In the latter case, the role of Ni–Si–P-enriched clusters is adopted by vacancy clusters.

**Figure 7:**
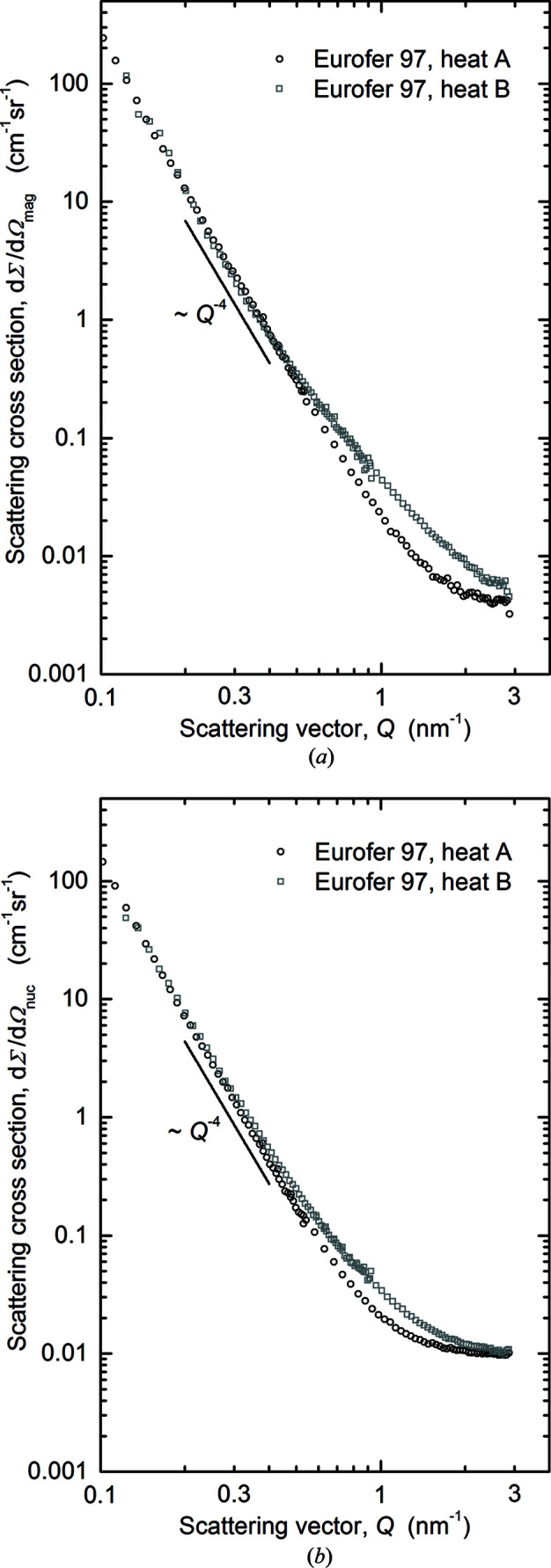
Scattering cross sections for the unirradiated conditions of heats A and B of Eurofer97: (*a*) magnetic scattering and (*b*) nuclear scattering.

**Figure 8:**
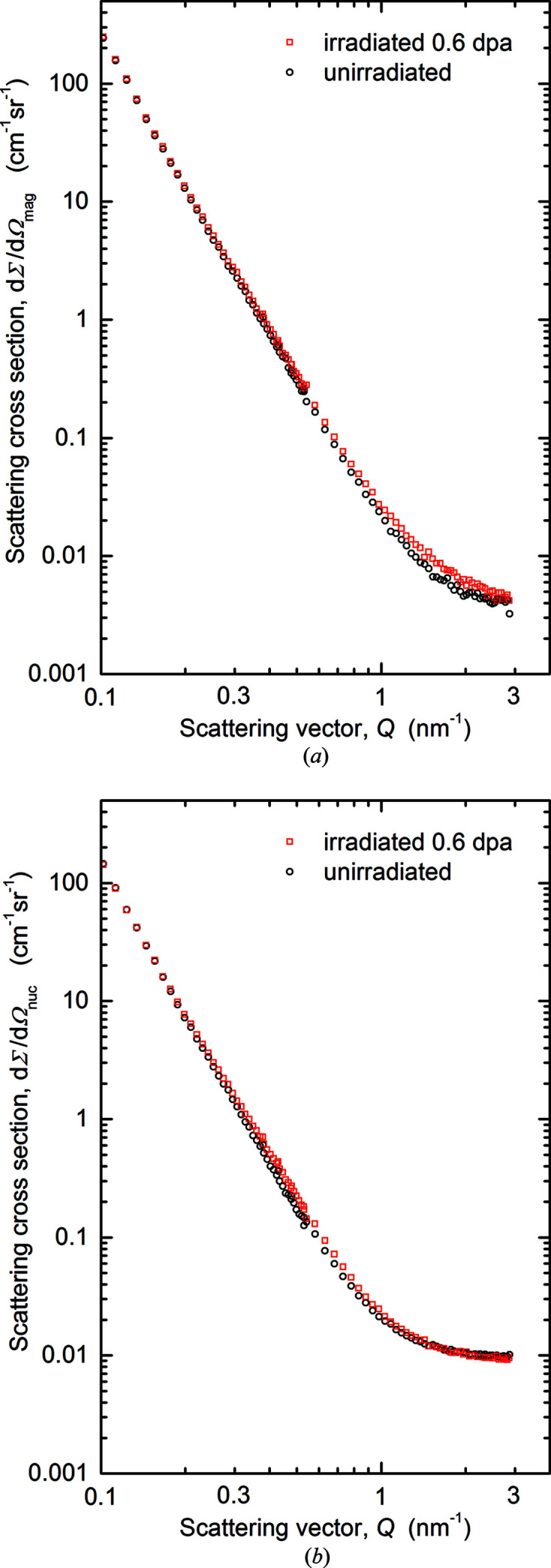
Scattering cross sections for the neutron-irradiated conditions (0.6 dpa) and the unirradiated reference of heat A of Eurofer97: (*a*) magnetic scattering and (*b*) nuclear scattering.

**Figure 9:**
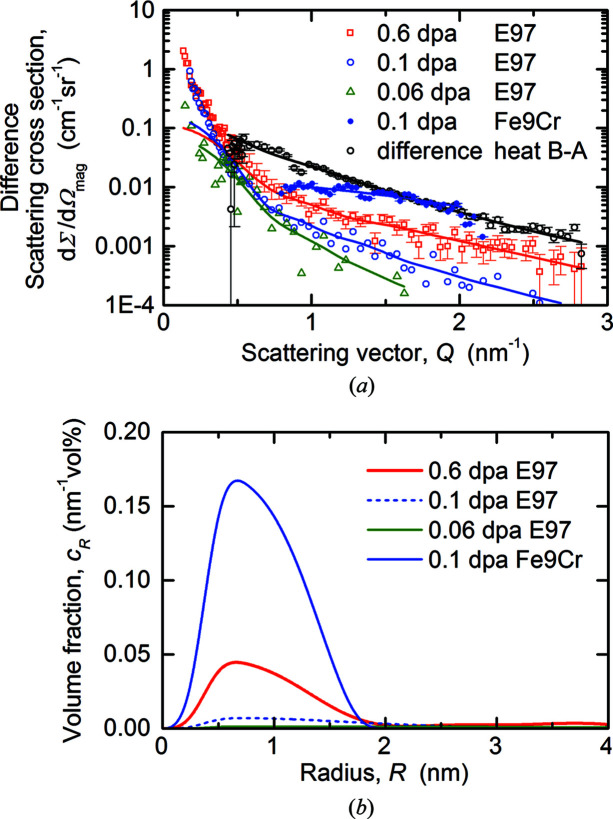
Magnetic difference scattering curves of Eurofer97 along with fits (*a*) and reconstructed size distribution of irradiation-induced scatterers for neutron-irradiated Eurofer97 (*b*). The black symbols on the left-hand side refer to the difference between heat B and heat A. Results obtained for a binary Fe–9Cr alloy (Konstantinović *et al.*, 2020 ▸) are included for comparison.

**Figure 10:**
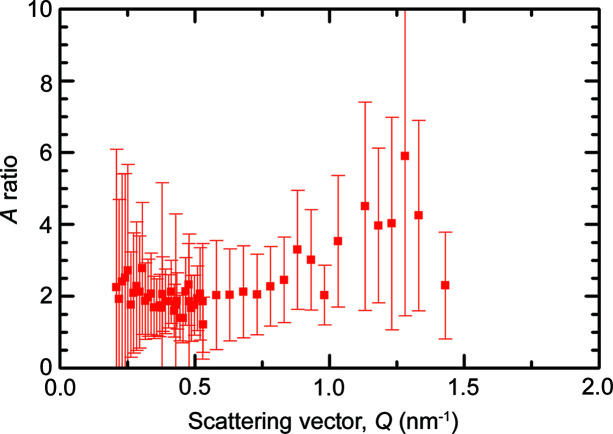
*A* ratio corresponding to the irradiation-induced difference scattering curves as function of *Q* for the 0.6 dpa irradiation of Eurofer97.

**Figure 11:**
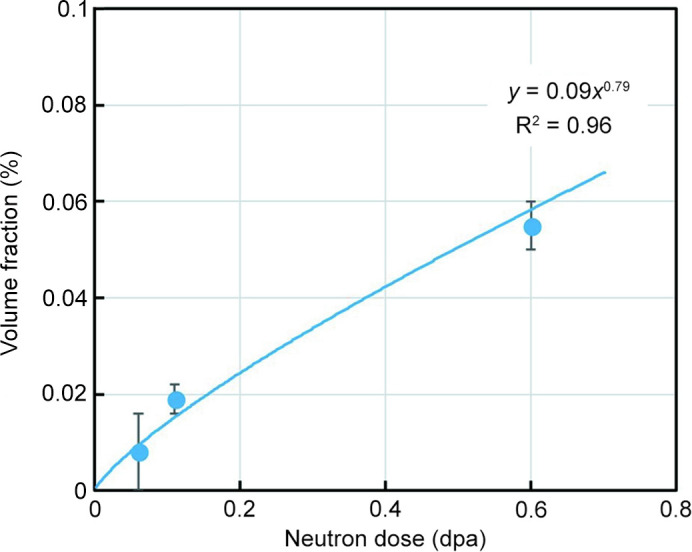
Volume fraction of scatterers as a function of neutron dose and best-fit power-law dependence. The volume fractions were derived from the measured magnetic scattering cross sections on the assumption of non-magnetic scatterers.

Finally, as indicated by the magnetic difference scattering curves in Figs. 7(*a*) ▸ and 9(*a*) ▸, the unirradiated heat B of Eurofer97 gives rise to significantly stronger scattering at higher *Q* values compared with unirradiated heat A. The difference is even larger than the increase due to neutron irradiation of heat A. This finding sheds further light on the remarkable irradiation resistance of Eurofer97. The source of this difference is beyond the scope of this study. The message here is rather to demonstrate that the irradiation effect at doses up to 0.6 dpa is weaker than the variability due to the fabrication process within specification limits.

## 5. Conclusions

In this study, characteristics of irradiation-induced nanofeatures derived from magnetic SANS have been reported for low-dose neutron-irradiated Fe–(5–14)Cr–NiSiP model alloys and the reduced-activation ferritic/martensitic 9Cr steel Eurofer97.

On the type of irradiation-induced scatterers, we conclude that Ni–Si–P-rich SRCs are the dominant scatterers in Fe–5Cr–NiSiP, while α′-phase particles dominate in Fe–14Cr–NiSiP, irradiated both at 290 and at 450°C. For Eurofer97 neutron-irradiated at 290°C up to 0.6 dpa, α′-phase particles and/or SRCs dominate.

The high volume fractions of α′-phase particles in irradiated Fe–14Cr–NiSiP give rise to interference effects in SANS and are consistent with the expectation based on the Fe–Cr binary equilibrium phase diagram with respect to the higher volume fraction at the lower irradiation temperature. The volume fraction of SRCs in irradiated (both temperatures) Fe–9Cr–NiSiP is much smaller, while the irradiation-induced hardness increase is only slightly smaller because of the higher obstacle strength of SRCs compared with α′-phase particles. The total volume fraction of scatterers deduced from SANS for irradiated Eurofer97 is still much smaller than for Fe–9Cr–NiSiP and even Fe–9Cr irradiated under the same conditions, which indicates an exceptionally high irradiation resistance of Eurofer97 in the dose range up to 0.6 dpa.

## Figures and Tables

**Figure 5:**
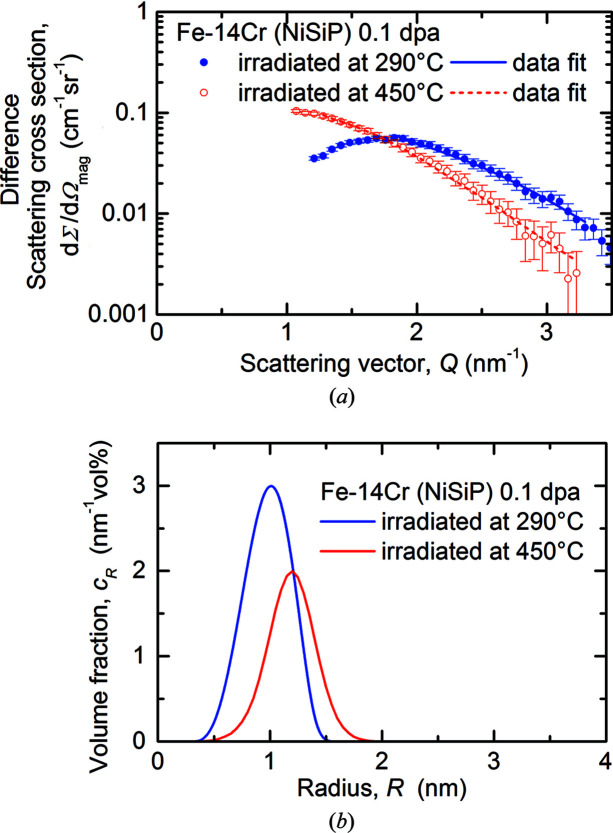
Magnetic difference scattering cross sections with fits (*a*) and size distributions (*b*) for Fe–14Cr–NiSiP.

**Table 1:** Composition of the Fe–Cr–NiSiP alloys in mass%

Alloy	Code	C	Cr	Ni	Si	P
Fe–5Cr–NiSiP	G384	<0.006	4.9	0.13	0.22	0.033
Fe–9Cr–NiSiP	G389	<0.006	9.1	0.092	0.22	0.032
Fe–14Cr–NiSiP	G394	<0.006	14.4	0.087	0.19	0.031

**Table 2:** Composition of heats A and B of Eurofer97 in mass%

Heat	Cr	C	Mn	V	W	Ta	P	S	B	Ti	Ni	Cu	Si
A	8.87	0.12	0.42	0.19	1.10	0.14	0.004	0.003	<0.0005	0.008	<0.007	0.022	0.07
B	8.82	0.11	0.47	0.20	1.09	0.13	0.005	0.004	<0.001	0.005	<0.02	0.016	0.04

**Table 3:** Average characteristics of scatterers, measured *A* ratios and measured Vickers hardness increase ΔHV10 for the irradiated Fe–Cr alloys

Material/irradiation	Volume fraction (%)	Mean radius (nm)	Width of size distribution (nm)	Average *A* ratio	ΔHV10
Fe–5Cr–NiSiP, 290°C	0.13±0.01	0.97±0.08	(0.2)–2.7	n.d.	68±5
Fe–5Cr–NiSiP, 450°C	0.16±0.01	1.49±0.07	0.5–3.9	n.d.	63±5
Fe–9Cr–NiSiP, 290°C†	0.29±0.01	0.95±0.05	(0.2)–1.7	1.95±0.15	72±3
Fe–14Cr–NiSiP, 290°C	1.75±0.05‡	0.83±0.08	0.5–1.4	2.05±0.2	94±7
Fe–14Cr–NiSiP, 450°C	1.10±0.05‡	1.07±0.08	0.6–1.7	2.1±0.3	79±6

† Konstantinović *et al.* (2020 ▸).

‡ Estimation based on the Porod invariant (Porod, 1982 ▸).

**Table 4:** Average characteristics of irradiation-induced scatterers and measured Vickers hardness increase ΔHV10 for the irradiated Eurofer97 samples

Irradiation	Volume fraction (%)	Mean radius (nm)	*A* ratio	ΔHV10
0.06 dpa, 290°C	0.008±0.008	Not determined	Not determined	0±6
0.11 dpa, 290°C	0.019±0.003	1.1±0.1	Not determined	6±5
0.6 dpa, 290°C	0.055±0.005	0.9±0.1	2.2±0.5	35±10
